# 靶向TROP-2的抗体药物偶联物在晚期非小细胞肺癌中的研究进展及展望

**DOI:** 10.3779/j.issn.1009-3419.2024.101.25

**Published:** 2024-10-20

**Authors:** Jingyan XU, Jiaqi LIU, Shiqi MEI, Qing ZHOU

**Affiliations:** 510080 广州，广东省肺癌研究所，南方医科大学附属广东省人民医院（广东省医学科学院）; Guangdong Provincial Institute of Lung Cancer, Guangdong Provincial People's Hospital Affiliated to Southern Medical University, Guangdong Academy of Medical Sciences, Guangzhou 510080, China

**Keywords:** 肺肿瘤, 可靶向基因组改变, 抗体药物偶联物, 人滋养层细胞表面抗原2, 药物治疗, Lung neoplasms, Actionable genomic alterations, Antibody-drug conjugates, Trophoblast cell surface antigen-2, Drug therapy

## Abstract

非小细胞肺癌（non-small cell lung cancer, NSCLC）仍是全球范围内的重大健康负担，患者亟待新的治疗选择。人滋养层细胞表面抗原2（trophoblast cell surface antigen-2, TROP-2）作为一种与NSCLC预后密切相关的靶点，已成为近年来的研究热点。其中，靶向TROP-2的抗体药物偶联物（antibody-drug conjugates, ADC）在NSCLC治疗领域取得了突破性进展。临床研究表明，部分TROP-2 ADC药物可显著改善既往经治的晚期/转移性NSCLC患者（无论是否伴有可靶向基因组改变）的无进展生存期，且在后线及一线治疗中均表现出可观的获益潜能。在药物安全性方面，尽管此类药物所引发的血液系统、呼吸系统和消化系统等各系统的不良反应大多可控，但仍需临床密切监测和及时管理。总之，TROP-2 ADC在NSCLC治疗领域具有广阔前景。

## Abstract (en)

Non-small cell lung cancer (NSCLC) remains a significant global health burden, and there is an urgent need for new treatment options. Trophoblast cell surface antigen-2 (TROP-2), a target closely associated with NSCLC prognosis, has become a research hotspot in recent years. Notably, TROP-2-targeted antibody-drug conjugates (ADCs) have made groundbreaking advances in NSCLC therapy. Clinical studies have demonstrated that certain TROP-2 ADCs can significantly improve progression-free survival in previously treated patients with advanced or metastatic NSCLC, regardless of the presence of actionable genomic alterations. These agents have shown promising potential in both frontline and subsequent treatment settings. In terms of safety, while adverse effects affecting the hematologic, respiratory, and gastrointestinal systems are generally manageable, close clinical monitoring and timely management are still required. In conclusion, TROP-2 ADCs hold great promise in the treatment of NSCLC.

肺癌是全球死亡率最高的肿瘤，其中85%为非小细胞肺癌（non-small cell lung cancer, NSCLC）^[1]^。中国约70%的NSCLC患者确诊时已进展至晚期，失去手术机会^[2]^，其疾病、社会和经济负担仍然沉重。尽管多年来随着免疫检查点抑制剂和靶向药物等的研发，NSCLC患者的生存期得以延长^[3]^，对于伴有可靶向基因组改变（actionable genomic alterations, AGAs）、基因突变的晚期NSCLC患者的治疗仍面临各种挑战，其在一线治疗耐药后缺乏有效治疗手段，后线化疗、化疗联合免疫疗法等疗效有限且面临毒副反应^[4]^；对于不伴有AGAs的NSCLC患者，一线化疗联合免疫标准治疗在程序性细胞死亡配体1（programmed death ligand 1, PD-L1）阳性率<1%或肺鳞癌患者群体中展现的疗效有限^[5]^，二线标准治疗，中位无进展生存期（progression-free survival, PFS）也仅有3-4个月^[6,7]^。因此，晚期/转移性NSCLC治疗仍存在大量未满足需求，并推动着新药/新型治疗方案的不断开发。

近年来有关抗体药物偶联物（antibody-drug conjugate, ADC）的研究不断带来突破。其中，肺癌领域备受关注的ADC靶点——人滋养层细胞表面抗原2（trophoblast cell surface antigen-2, TROP-2）能在肺癌组织内高度表达，参与肿瘤发展的多种信号通路，且与肺癌预后密切相关^[8]^。目前已进入临床试验阶段治疗NSCLC的TROP-2 ADC包括戈沙妥珠单抗^[9]^、Datopotamab Deruxtecan（Dato-DXd）^[10]^和SKB264^[11]^等数十种。本文将针对治疗NSCLC患者的TROP-2 ADC的研究新进展进行综述，以期加深临床医生对此类药物的了解。

## 1 TROP-2在NSCLC中的表达及作用

TROP-2可在NSCLC、乳腺癌、结直肠癌等多种肿瘤细胞中高表达，具有调节细胞生长、转化和增殖等功能^[12]^。在NSCLC中，64%的肺腺癌和75%的鳞状细胞癌均观察到TROP-2的高表达^[8]^。TROP-2通路通过钙信号和β-catenin信号以及胞质内蛋白激酶C受体1（receptor for activated C kinase 1, RACK1）的重新定位参与促进血管生成、细胞生长和转移。具体机制涉及：TROP-2分子胞质结构域可被蛋白激酶C（protein kinase C, PKC）磷酸化，导致Ca^2+^的释放，进而激活酪氨酸/丝氨酸蛋白激酶（Raf）通路和核因子-κB（nuclear factor kappa B, NF-κB）通路，刺激丝裂原活化蛋白激酶（mitogen-activated protein kinase, MAPK）信号传导和细胞周期的进展，诱导细胞内的转录激活因子（activator protein 1, AP-1）调控多个肿瘤相关靶基因^[13]^。TROP-2胞内结构域可通过β-catenin信号传导，促进干细胞/祖细胞的自我更新^[14]^。TROP-2还可通过激活整合素β1-RACK1-FAK-Src通路，降低肿瘤细胞的黏附作用，促进肿瘤侵袭和转移^[15]^。因此，TROP-2不仅是一个肿瘤标志物，更可能在NSCLC的发生和进展中起到重要作用。

基于上述机制，针对TROP-2靶点的药物陆续被研发，近期的一些研究进一步支持了TROP-2与NSCLC的关系。TROP-2在NSCLC中的高表达与患者预后密切相关。2023年美国临床肿瘤学会（American Society of Clinical Oncology, ASCO）年会报告了一项基于全球最大NSCLC基因数据集的分析^[16]^，发现TROP-2高表达组NSCLC患者的PFS（P<0.001）和总生存期（overall survival, OS）（P=0.007）均较低表达组更短；且无论PD-L1的表达如何，TROP-2高表达均与较差的预后独立相关。此外，一项临床前研究^[17]^在2024年美国癌症研究协会年会中报告，靶向TROP-2的ADC类药物Dato-DXd对颅内TROP-2表达阳性NSCLC小鼠模型具有显著抗肿瘤活性，且对比同种型对照IgG-ADC具有显著的生存获益（P=0.0002），从而支持Dato-DXd针对中枢神经受累患者的治疗。因此，TROP-2在NSCLC中的潜在临床价值，尤其是在ADC药物的开发中，已成为当前研究的焦点。

## 2 TROP-2 ADC的构成及作用机制

ADC主要由单克隆抗体（后文简称单抗）、有效载荷和连接体三部分所构成^[18]^，图1展示了TROP-2 ADC的构成组分及作用机制。目前NSCLC治疗领域正在研发的TROP-2 ADC中，戈沙妥珠单抗、SKB264和Dato-DXd均已进入III期临床试验阶段，表1总结了此3种药物的构成及靶向机制特征。

**图1:**
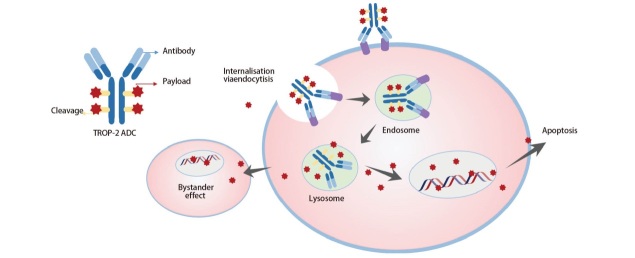
TROP-2 ADC的结构及作用机制

**表1:** 治疗NSCLC的3种TROP2-ADC的组成及主要特征

	Sacituzumab govitecan^[19]^	Dato-DXd^[10]^	SKB264^[11,39]^
mAb	hRS7	hTINA1	hRS7
Payload	SN-38	DXd	KL610023
Linker	CL2A linker	Tetrapeptide-based cleavable linker	Sulfonyl pyrimidine-CL2A-carbonate linker
Cleavage mode	pH mediated	Enzyme mediated	pH mediated
K_D_ (nmol/L)	0.2935	0.74	0.3083
DAR	7.60±0.03	4	7.4
Bystander effect	Yes	Yes	Yes
Tumor growth inhibition* (%)	21.05	96	123.47

K_D_: dissociation constant; DAR: drug-to-antibody ratio. *: Based on HCC1806 xenograft model or NCI-N87 xenograft model.

戈沙妥珠单抗由抗TROP-2单抗Sacituzumab与拓扑异构酶I抑制剂伊立替康的活性代谢物（SN-38）通过可切割的CL2A连接体共轭构成^[19]^。CL2A连接体对pH敏感，约48 h后戈沙妥珠单抗在动物和人血浆中释放的载荷几乎达到100%^[11]^。Sacituzumab是一种高亲和力的人源化IgG1，可通过结合Fc受体而诱导抗体依赖性细胞介导的细胞毒性等功能^[19]^。SKB264与戈沙妥珠单抗具有相同的单抗组分和相似的作用机制，该药采用了含有对pH敏感的2-（甲磺酰基）嘧啶接头的连接体，将抗体与载荷贝洛替康衍生物拓扑异构酶I抑制剂（KL610023）共轭连接；这种连接体的稳定性较高，体外研究^[11]^显示约144 h后SKB264于血浆中释放的载荷可达到70%。相较于戈沙妥珠单抗，相同条件下SKB264在有效载荷暴露量、半衰期和肿瘤抑制率等方面均优于戈沙妥珠单抗^[11]^。

Dato-DXd由抗TROP-2 IgG1单抗Datopotamab与拓扑异构酶I抑制剂依喜替康衍生物（exatecan derivate, DXd）通过可裂解的四肽连接体偶联构成^[10]^。该药的连接体通过特异性酶促裂解释放药物，具有极高的稳定性，可以实现对TROP2阳性肿瘤细胞的精准杀伤^[10]^。Dato-DXd将药物抗体比值（drug-to-antibody ratio, DAR）优化为4，平衡了药物的疗效和毒性，最大化治疗窗口^[10]^。此外，由于已释放的游离载荷可继续发挥“旁观者效应”（图1），如果其半衰期过长也可能使游离载荷进入循环带来系统性毒性。有研究^[20]^显示，游离DXd的半衰期仅1.37 h，这保证Dato-DXd具备旁观者效应而不增加过多系统性毒性。

总的来说，TROP-2 ADC药物的开发为NSCLC治疗提供了一个崭新的方向。近年来，戈沙妥珠单抗、Dato-DXd和SKB264等TROP-2 ADC已在NSCLC治疗领域展现出初步的疗效和可控的安全性，具有广阔的应用前景。

## 3 TROP-2 ADC在晚期NSCLC中的临床疗效

### 3.1 戈沙妥珠单抗

作为首个上市的TROP-2 ADC，戈沙妥珠单抗目前在美国已获批了尿路上皮癌、三阴性乳腺癌和激素受体（hormonal receptor, HR）阳性/人表皮生长因子受体2（human epidermal growth factor receptor 2, HER2）阴性乳腺癌的适应证。

近年来，戈沙妥珠单抗治疗NSCLC的研究也获得了阶段性成果。IMMU-132-01研究^[9]^纳入了接受过多线治疗的晚期实体瘤患者（10.9% NSCLC），其中NSCLC队列结果^[9]^显示，既往经治合并转移的晚期NSCLC患者接受戈沙妥珠单抗治疗后的客观缓解率（objective response rate, ORR）达到了16.7%，中位PFS和OS分别达到4.4和7.3个月。EVOKE-01研究^[21]^评估了戈沙妥珠单抗与多西他赛在铂类化疗、抗程序性死亡受体1（programmed cell death 1, PD-1）/PD-L1和AGAs靶向治疗期间或治疗后进展的转移性NSCLC患者中的疗效，其初步分析显示研究未达到OS的主要终点，与多西他赛组相比，戈沙妥珠单抗组显示mOS具体数值的改善（11.1 vs 9.8个月，HR=0.84，95%CI：0.68-1.04），在鳞状和非鳞状亚组中结果一致（表2）。EVOKE-02研究^[22,23]^发现未经治疗的转移性NSCLC患者在接受戈沙妥珠单抗联合帕博利珠单抗治疗后，其中PD-L1肿瘤比例评分（tumor proportion score, TPS）≥50%和TPS<50%的患者均可得到可观的早期缓解（表2）。这些结果提示了TROP-2 ADC对于治疗NSCLC患者的潜力和广阔前景。

**表2:** TROP-2 ADC治疗晚期NSCLC/实体瘤的临床试验结果汇总

Study/Author (Time of publication)	Phase & design	Population	Intervention	Efficacy endpoints				
				ORR	DCR/CBR	mOS (mon)	mPFS (mon)	mDOR (mon)
IMMU-132-01 (2021)^[9]^	Phase I/II, cohort study	Advanced or metastatic solid tumors that relapsed (or refractory) after standard therapy (10.9% NSCLC)	Sacituzumab govitecan	NSCLC cohort: 16.7%	NSCLC cohort CBR: 24.1%	NSCLC cohort: 7.3	NSCLC cohort: 4.4	NSCLC cohort: 6.0
EVOKE-01 (2024)^[21]^	Phase III, RCT	Metastatic NSCLC with progression on/after platinum-based chemotherapy, anti-PD-1/PD-L1, and targeted treatment for AGAs	Sacituzumab govitecan/ Docetaxel	13.7% (Sacituzumab govitecan) vs 18.1% (Docetaxel)	DCR: 67.6% (Sacituzumab govitecan) vs 67.1% (Docetaxel)	11.1 (Sacituzumab govitecan) vs 9.8 (Docetaxel)	4.1 (Sacituzumab govitecan) vs 3.9 (Docetaxel)	6.7 (Sacituzumab govitecan) vs 5.8 (Docetaxel)
EVOKE-02 (2023)^[22,23]^	Phase II, RCT	Untreated metastatic NSCLC, without AGAs	Sacituzumab govitecan + Pembrolizumab ± Platinum	Cohort A (PD-L1 TPS≥50%)-total: 67% (squamous, 67% vs non-squamous, 67%) Cohort B (PD-L1 TPS< 50%): 44%, DCR: 78%	/	/	Cohort A total: 13.1 (squamous, not reached vs non-squamous, 13.1)	Cohort A: not reached
TROPION-PanTumor01 (2023)^[24]^	Phase I, dose-escalation/expansion study	Relapsed/progressed after standard therapy (or no standard therapy is available), advanced or metastatic NSCLC with or without AGAs	Dato-DXd	6 mg/kg cohort: 26%	/	6 mg/kg cohort: 11.4	6 mg/kg cohort: 6.9	6 mg/kg cohort: 10.5
TROPION-PanTumor02 (2024)^[26]^	Phase I/II, single arm	Advanced or metastatic NSCLC with or without AGAs	Dato-DXd	Total: 45% (squamous, 29.4% vs non-squamous, 56.5%)	/	/	Total PFS: 7.4 (squamous, 5.5 vs non-squamous, 9.6)	5.6
TROPION-Lung01 (2024)^[27]^	Phase III, RCT	Patients with previously treated advanced or metastatic NSCLC, with or without AGAs, AGAs negative patients received platinum-based chemotherapy and anti-PD-1/PD-L1 treatment; Patients with AGAs deteriorated after one platinum-based therapy and 1/2 line of targeted therapy	Dato-DXd/ Docetaxel	Total: 79% (Dato-DXd) vs 39% (Docetaxel); squamous, 6% vs 9%; non-squamous, 73% vs 30%	Total DCR: 77.3% (Dato-DXd) vs 64.9% (Docetaxel) squamous, 66.2% vs 77.5%; non-squamous, 80.3% vs 61.1%	Total: 12.9 (Dato-DXd) vs 11.8 (Docetaxel) squamous, 7.6 vs 9.4; non-squamous, 14.6 vs 12.3 (AGA: 15.6 vs 9.8)	Total: 4.4 (Dato-DXd) vs 3.7 (Docetaxel) squamous, 2.8 vs 3.9; non-squamous, 5.5 vs 3.6 (AGA: 5.7 vs 2.6)	Total: 7.1 (Dato-DXd) vs 5.6 (Docetaxel); squamous, 5.9 vs 8.1; non-squamous, 7.7 vs 5.6 PFS: 4.4 (Dato-DXd)/3.7 (docetaxel)
TROPION-Lung02 (2024)^[32,33]^	Phase Ib, dose-escalation and -expansion study	Advanced NSCLC with AGAs negative	Dato-DXd plus Pembrolizumab ± Platinum	1L subgroup: Total: 52% (Doublet) vs 56% (Triplet); PD-L1 TPS≥50%, 100% vs 53%; PD-L1 TPS<50%, 46% vs 56%	1L subgroup: Total DCR: 88% (Doublet) vs 89% (Triplet); PD-L1 TPS≥50%, 100% vs 87%; PD-L1 TPS<50%, 86% vs 90%	/	1L subgroup: Total: 11.1 (Doublet) vs 6.8 (Triplet); PD-L1 TPS≥50%, NE vs 6.8; PD-L1 TPS<50%, 9.3 vs 6.8	1L subgroup: Total: NE (Doublet) vs 12.9 (Triplet); PD-L1 TPS≥50%, NE vs 18.1; PD-L1 TPS<50%, 12.0 vs 12.9
TROPION-Lung04^[34,43]^ (2023)	Phase Ib, cohort study	Patients with advanced or metastatic NSCLC, AGAs negative, treatment-naive or had ≤1 prior line of systemicchemotherapy without concomitant ICIs	Dato-DXd plus Duvalumab ± Platinum	50% (Doublet) vs 76.9% (Triplet)	DCR: 92.9% (Doublet) vs 92.3% (Triplet)	/	/	/
TROPION-Lung05 (2023)^[24,29,31]^	Phase II, single arm	Advanced or metastatic NSCLC with AGAs, at least one prior platinum-based chemotherapy and ≥1 line of targeted therapy	Dato-DXd	Total: 35.8%, EGFR mutant: 43.6% Subgroup: Asian cohort: 42.4% EGFR mutant: 48.9% Subgroup: brain metastases vs without brain metastases: 28% vs 40%	Total DCR: 78.8% Subgroup: Asian cohort DCR: 80.3% Subgroup: brain metastases vs without brain metastases DCR: 72% vs 83%; CBR: 40% vs 51%	/	Subgroup: brain metastases vs without brain metastases: 5.4 vs 5.6	Total: 7.0 Subgroup: Asian cohort: 4.4
ICARUS-LUNG01 (2024)^[28]^	Phase II, single arm	Metastatic, unresectable NSCLC with progressive disease after 1 to 3 lines of standard therapy	Dato-DXd	Total: 28% (squamous, 5.0% vs non-squamous, 32.9%)	DCR: 75%	Total: 11.9 (squamous, 6.3 vs non-squamous, 12.6)	Total: 3.6 (squamous, 2.9 vs non-squamous, 4.8)	/
Fang, et al. (2023)^[35,39]^	Phase II, cohort study	Relapsed or refractory locally advanced/metastatic NSCLC and other tumors	SKB264	Total: 43.6%, EGFR mutant: 60% EGFR wild: 26.3% (non-squamous, 22.2%)	/	Total: 22.6, EGFR mutant: 22.7 EGFR wild: 14.1 (non-squamous, 16.2)	Total: 7.2, EGFR mutant: 11.5 EGFR wild: 5.3 (non-squamous, 5.8)	Total: 9.3, EGFR mutant: 8.7 EGFR wild: 9.6
OptiTROP-Lung01 (2024)^[36]^	Phase II, cohort study	Treatment naive advanced NSCLC without actionable genomic alterations	SKB264 5 mg/kg Q3W plus KL-A167 1200 mg Q3W (cohort 1A) SKB264 5 mg/kg Q2W plus KL-A167 900 mg Q2W (cohort 1B)	Cohort 1A: 48.6% Cohort 1B: 77.6%	Cohort 1A: 94.6% Cohort 1B: 100%	/	Cohort 1A: 15.4% Cohort 1B: not reached	/
King, et al. (2018)^[37]^	Phase I, single arm	Untreated advanced or metastatic solid tumors (19% NSCLC)	PF-06664178	SD: 37.9% (n=11; NSCLC: n=0), no PR or CR	/	/	/	/
Marathe O, et al. (2023)^[38]^	Phase I/II, single arm	Advanced/metastatic solid tumors that failed standard therapy	DB-1305	NSCLC cohort: 46.2%	NSCLC cohort DCR: 92.3%	/	/	/2.3% (12/13)
Jie W (2023)^[44]^	Phase I, single arm	Advanced malignant solid tumors	SHR-A1921	PR: 10/38 (NSCLC=5)	/	/	/	/

NSCLC: non-small cell lung cancer; RCT: randomized controlled trial; ORR: objective response rate; CR: complete response; PR: partial response; mDOR: median duration of response; DCR: disease control rate; mOS: median overall survival; mPFS: median progression-free survival; SD: stable disease; CBR: clinical benefit rate; TPS: tumor proportion score; AGAs: actionable genomic alterations; PD-1: programmed cell death 1; PD-L1: programmed death ligand 1; ICI: immune checkpoint inhibitor; EGFR: epidermal growth factor receptor.

### 3.2 Dato-DXd

TROPION-PanTumor01研究^[24]^纳入了伴或不伴AGAs的、经标准治疗后进展或复发的晚期/转移性NSCLC患者。Dato-DXd 6 mg/kg在该研究中初步展现出抗肿瘤活性：患者ORR值达到26%，中位PFS和OS分别达到6.9和11.4个月（表2）^[24]^。2022年Shimizu等^[25]^报告该研究日本队列亚组患者在接受Dato-DXd（4-8 mg/kg）治疗后的ORR值也达到了26%。在中国进行的TROPION-PanTumor02研究^[26]^中，Dato-DXd 6 mg/kg在晚期或转移性NSCLC队列也显示出一定疗效，其中非鳞状细胞亚组在ORR和PFS方面获益更大（表2）。

TROPION-Lung01研究^[27]^是首个在肺癌领域获得阳性结果的III期TROP-2 ADC研究，结果显示相较于多西他赛，接受Dato-DXd治疗能够显著改善伴或不伴AGAs的晚期/转移性NSCLC患者的PFS达25%（P=0.004），并表现出更好的长效缓解。非鳞癌NSCLC亚组人群中，Dato-DXd单药相比多西他赛治疗均使患者的PFS显著延长（5.5 vs 3.6个月），其中AGAs亚组也显示Dato-DXd单药相比多西他赛治疗能更好地延长患者的PFS和OS^[27]^。另一项Dato-DXd在NSCLC患者中的单臂研究ICARUS-LUNG01^[28]^初步报告了与TROPION-Lung01研究相似的疗效（表2），该研究在未来将进行多项探索性分析以确定与疗效/耐药相关的生物标志物。II期TROPION-Lung05试验^[24,29,30]^纳入了仅伴有AGAs的晚期/转移性NSCLC患者，发现接受Dato-DXd单药治疗的总体ORR为35.8%，其中占比最大的表皮生长因子受体（epidermal growth factor receptor, EGFR）突变患者的ORR达到43.6%；该研究中亚洲队列报告的ORR为42.4%，其中EGFR突变患者的ORR达到了48.9%。该研究在2024年ASCO年会上报告了颅内疗效分析，研究共纳入53例基线时存在稳定脑转移灶的患者，占研究患者总数的39%，其中55%的患者为EGFR突变阳性^[31]^。在具有靶病灶的18例患者中，颅内ORR达到22%，中位颅内起效时间为1.5个月，中位颅内缓解持续时间为5.5个月^[31]^。此外，研究发现无论基线时是否伴有脑转移，患者均可从治疗中获益（表2），这表明Dato-DXd治疗对脑转移患者具有潜在的疗效^[31]^。

在联合方案探索中，2024年ASCO年会上报告了Ib期TROPION-Lung02研究^[32,33]^的亚组结果：作为初治或经治的AGAs阴性晚期/转移性NSCLC患者的一线治疗方案，Dato-DXd联合帕博利珠单抗双联组和在此基础上加铂类化疗的三联组患者的ORR值分别达到52%和56%。另一项Ib期TROPION-Lung04研究^[29,34]^报告了Dato-DXd联合度伐利尤单抗±铂类也可在初治或经治的AGAs阴性晚期/转移性NSCLC患者中表现出良好的治疗反应（表2）。

这些数据提示了，无论作为一线治疗或二线/后线治疗，Dato-DXd单药或联合方案对于晚期伴或不伴转移的NSCLC患者均有显著疗效，尤其是作为一线治疗，且该药具有可耐受的安全性。未来随着III期TROPION-Lung07、08和AVANZAR等研究（表3）结果的公布，将会为NSCLC患者提供更多Dato-DXd治疗方案的选择。

**表3:** 目前正在NSCLC患者中开展的TROP-2 ADC相关临床试验

Study name/registration number	Phase and design	Population	Intervention	Study status
TROPiCS-03	Phase II, single arm	Metastatic solid tumors (containing NSCLC, NSCLC patients progress after platinum-based chemotherapy and anti-PD-1/PD-L1 therapy)	Sacituzumab govitecan	Active, not recruiting
EVOKE-03	Phase III, RCT	PD-L1 TPS≥50%, metastatic NSCLC, no prior anti-PD-1/PD-L1 therapy	Sacituzumab govitecan plus Pembrolizumab/Pembrolizumab	Recruiting
VELOCITY-Lung	Phase II, RCT	Advanced or metastatic NSCLC, AGAs patients with no targeted therapy	Multi-agent combination therapy (including Sacituzumab govitecan)	Recruiting
Morpheus-Lung	Phase I/II, RCT	Metastatic NSCLC, cohort 1 without systemic treatment, PD-L1 TPS≥50%; Cohort 2 progressed after prior platinum-based and anti-PD-L1/PD-1 therapy	CIT combination therapy (including Sacituzumab govitecan)	Active, not recruiting
TROPION-Lung07	Phase III, RCT	PD-L1 TPS<50% advanced or metastatic NSCLC, with no prior therapy, AGAs negative	Dato-DXd plus Pembrolizumab/Pembrolizumab plus Pemetrexed±Platinum	Recruiting
TROPION-Lung08	Phase III, RCT	PD-L1 TPS≥50% in treatment-naive advanced or metastatic NSCLC, AGAs negative	Dato-DXd in combination with Pembrolizumab/Pembrolizumab	Recruiting
TROPION-Lung10	Phase III, RCT	PD-L1 TPS≥50% in treatment-naive advanced or metastatic non-squamous NSCLC, AGAs negative	Dato-DXd in combination with Rilvegostomig or Rilvegostomig monotherapy vs Pembrolizumab monotherapy	Recruiting
TROPION-Lung14	Phase III, RCT	Advanced/metastatic NSCLC harboring an EGFR-sensitizing mutation	Dato-DXd plus Osimertinib vs Osimertinib	Recruiting
TROPION-Lung15	Phase III, RCT	EGFR mutation locally advanced or metastatic NSCLC whose disease has progressed on prior Osimertinib treatment	Dato-DXd ± Osimertinib vs Platinum-based doublet chemotherapy	Not yet recruiting
AVANZAR	Phase III, RCT	Advanced or metastatic NSCLC without first-line chemotherapy or other systemic therapy, AGAs negative	Dato-DXd plus Duvalumab plus Platinum/Pembrolizumab plus Platinum	Recruiting
ORCHARD	Phase II, cohort study	Advanced or metastatic EGFR-mutated NSCLC that progressed after first-line treatment with Osimertinib	Multi-regimen therapy with Dato-DXd plus Osimertinib	Active, not recruiting
NCT05870319	Phase III, RCT	Advanced or metastatic NSCLC with EGFR mutation but EGFR-TKI treatment failed	SKB264 monotherapy/Pemetrexed plus Platinum treatment	Recruiting
MK-2870-007	Phase III, RCT	PD-L1 TPS≥50% in metastatic NSCLC	SKB264 in combination with Pembrolizumab/Pembrolizumab	Recruiting
NCT05949619	Phase I/II, single arm	Advanced or metastatic NSCLC or other solid tumors	BL-M02D1	Not yet recruiting

CIT: cancer immunotherapy; TKI: tyrosine kinase inhibitor; RP2D: recommended dose for phase II clinical trials.

### 3.3 SKB264

一项针对复发或难治性局部晚期/转移性实体瘤患者的II期研究^[35]^公布了其中NSCLC队列的结果，发现接受SKB264治疗的患者ORR值达到了43.6%，中位缓解持续时间为9.3个月。其中EGFR野生型亚组（既往接受包括抗PD-1/PD-L1在内的2线治疗）的ORR为26.3%，中位PFS为5.3个月，中位OS为14.1个月；而对于酪氨酸激酶抑制剂（tyrosine kinase inhibitors, TKIs）耐药的EGFR突变型NSCLC（50%的患者至少经历1次化疗失败）亚组患者，ORR可达60%，中位PFS为11.5个月（表2）^[35]^。另有一项II期研究^[36]^在初治且不伴AGA改变的晚期NSCLC患者中报告了使用SKB264联合KL-A167（抗PD-L1）的初步疗效。研究探索了不同剂量和给药间隔组合，在两组队列中均显示出良好的治疗反应（表2）。特别是在SKB264 5 mg/kg合并KL-A167 900 mg（每2周给药1次）队列中，鳞状与非鳞状NSCLC患者ORR分别达到了84.0%和72.5%，6 个月PFS率分别为73.5%和93.8%；PD-L1表达为1%、1%-49%和≥50%的患者ORR分别达到63.2%、81.3%及87.0%，6个月PFS率分别为82.2%、76.6%和91.3%^[36]^。

在上述研究基础上，目前III期SKB264-III-09试验正在积极推进中，该研究将在伴EGFR或其他基因突变但EGFR-TKIs治疗失败的晚期/转移性NSCLC患者中，对比SKB264单药和培美曲塞联合铂类药物的疗效和安全性。此外，正在招募患者的III期MK-2870-007研究也将于PD-L1 TPS≥50%的转移性NSCLC患者的一线治疗中进行SKB264联合帕博利珠单抗与帕博利珠单抗单药的疗效与安全性比较（表3）。

### 3.4 其他正在开展的TROP-2

ADC临床研究 表3总结了目前正在积极开展中的TROP-2 ADC治疗NSCLC（包括含有NSCLC队列的实体瘤）患者的临床试验，其中大多数研究的主要疗效终点设计为ORR、PFS或OS，安全性终点旨在观察不良事件、治疗期出现的不良事件或严重不良事件的发生。

已在实体瘤患者的NSCLC队列人群中获得了初步探索结果的TROP-2 ADC尚有PF-06664178和DB-1305（表2）。PF-06664178是一种通过靶向TROP-2将细胞毒性药物Aur0101递送至肿瘤细胞的ADC，其I期研究纳入了31例（19%）晚期或转移性NSCLC患者并获得了37.9%的最佳总体反应（病情稳定的患者比例），然而该研究因毒性过大而提前结束^[37]^。DB-1305是一种通过四肽将抗TROP-2抗体与新型拓扑异构酶I抑制剂P1021偶联而成的靶向ADC；有研究者^[38]^在2023年欧洲肿瘤内科学会会议上报告了DB-1305的I/II期单臂临床试验的初步结果，发现该药治疗既往接受过标准治疗而失败的NSCLC队列患者的ORR达到了46.2%。另一种TROP-2 ADC，BL-M02D1的有效载荷为小分子毒素Ed-04（拓扑异构酶抑制剂），其I/II期临床试验也已经启动，旨在晚期/转移性NSCLC或其他实体肿瘤中探索该药的安全性、最佳剂量和初步疗效（表3）。

## 4 TROP-2 ADC在晚期NSCLC中的安全性及其管理

ADC研发追求药物毒性和有效性的平衡，优化抗体、有效载荷和连接体一直是ADC药物的前沿问题。降低不良反应和实现不良反应的可管理是ADC临床应用的重点。

在IMMU-132-01研究^[9]^中，患者接受戈沙妥珠单抗治疗后发生治疗相关消化系统不良反应最常见有恶心和腹泻，分别占总患者人群的62.6%和56.2%，多数为轻微反应，3级恶心和腹泻发生率分别为3.6%和7.9%；治疗相关血液系统不良反应最常见的有中性粒细胞减少（57.8%），3级中性粒细胞减少和发热性中性粒细胞减少的发生率分别为42.4%和5.3%^[9]^。EVOKE-01研究^[21]^报告戈沙妥珠单抗和多西他赛组分别有6.8%和14.2%的患者因治疗相关不良事件而停药，其中1.4%和1.0%导致死亡。戈沙妥珠单抗组中性粒细胞减少和≥3级中性粒细胞减少发生率分别为37.5%和24.7%，低于多西他赛组的42.7%和36.8%^[21]^。

在TROPION-PanTumor01研究^[24]^中，Dato-DXd治疗引起的任何级别中性粒细胞计数减少的发生率为5.6%，且大多数中性粒细胞减少症为1或2级，没有出现≥4级的中性粒细胞减少症或任何级别的发热性中性粒细胞减少症。在TROPION-Lung01研究^[27]^中，Dato-DXd较传统化疗多西他赛的血液学毒性明显降低，≥3级中性粒细胞减少症发生率为0.7%（多西他赛组为23.4%），且暂未报告其他显著血液学毒性事件。对于间质性肺病（interstitial lung disease, ILD），目前Dato-DXd相关临床试验所报告的ILD事件大多为1/2级，多数经治疗后可控制病情。TROPION-PanTumor01研究^[24]^报告了接受6 mg/kg Dato-DXd治疗的NSCLC患者中有3例（6%）发生与药物相关的ILD（2例为2级，1例为4级）；TROPION-Lung01研究^[27]^报告了使用6 mg/kg Dato-DXd的NSCLC患者有3.7%发生≥3级的药物相关的ILD或肺炎，多西他赛组的比例为1.4%。

SKB264目前可获取的临床研究安全性资料较少，在血液系统毒性方面，有研究^[35,39]^报告了治疗相关的≥3级中性粒细胞计数下降和贫血分别占患者人群的34.9%和30.2%，≥3级白细胞计数下降的发生率为25.6%，≥3级的口腔炎发生率为9.3%。

总体上，大多数TROP-2 ADC治疗NSCLC的安全性可控，相关不良反应可基于国内外抗肿瘤治疗相关指南/专家共识，结合临床诊治经验进行相应处理（表4）。

**表4:** TROP-2 ADC的常见安全性问题及管理方法

Types of adverse reactions	Clinical features	Recommend management methods
Blood system	Thrombocytopenia, neutropenia	⋅ For thrombocytopenia, routine platelet boosting therapy is required^[45]^. ⋅ For neutropenia, granulocyte colony-stimulating factor can be used as a routine drug. For patients with neutropenia and fever or presence of recurrent infection, empirical antibiotic therapy must be immediately selected according to the patient’s risk level^[46]^.
Pulmonary	Interstitial lung disease/Noninfectious pneumonia	⋅ For grade 3 or higher pulmonary toxicity, the drug should be discontinued permanently, and empiric high-dose methylprednisolone therapy should be started immediately^[46]^. ⋅ For the treatment of interstitial lung disease/non-infectious pneumonia, please refer to the expert consensus on the diagnosis and treatment of anticancer drug-induced interstitial lung disease^[47]^.
Digestive system	Nausea, vomiting, diarrhea, severe complications such as intestinal perforation, erosion, ulcer	⋅ For grade 3 or 4 gastrointestinal adverse reactions, pause ADC treatment until it regresses to ≤grade 1, reduce the dose of ADC upon re-administration, and provide supportive therapy such as antiemetics and antidiarrheals. Consider treatment discontinuation in case of repeated grade≥3 gastrointestinal adverse reactions^[46]^.
Stomatitis	Mouth pain, mouth ulcers and erythema	⋅ Local symptomatic treatment is the main treatment, and use systemic treatment for auxiliary treatment^[48]^.
Ocular toxicity	Keratitis, conjunctivitis, dry eye and ulcerative keratitis	⋅ For grade 2 or 3 adverse events, treatment should be paused. Autologous serum eye drops can be used to alleviate symptoms, followed by a reduction of one dose level upon improvement for treatment continuation. And treatment should be discontinued for grade 4^[49]^.

## 5 总结与展望

尽管肺癌治疗手段不断丰富，临床上针对NSCLC的一线至后线治疗的需求仍未得到满足，例如AGAs人群的一线治疗需考虑分子特征、病理学变异和临床表现，不伴AGAs人群在一线治疗中由于缺乏充分的分子标志物指导，且后线治疗患者的生存期仍较短^[40]^。目前，TROP-2 ADC在NSCLC领域的研究正在不断深入，无论是在前线治疗、后线治疗，还是在不同疾病亚型，都已经展开了大量临床实验，提示TROP-2 ADC在NSCLC治疗中具有潜力，并值得进一步关注和探索。

### 5.1 后线治疗

在后线治疗领域，戈沙妥珠单抗和Dato-DXd分别展开了与多西他赛的头对头比较。戈沙妥珠单抗相关的3期EVOKE-01研究在转移性NSCLC患者中仅显示mOS具体数值的改善，未达到OS的主要终点。Dato-DXd相关的TROPION-Lung01研究取得了阳性成果，能够显著改善伴或不伴AGAs的晚期或转移性NSCLC患者的PFS和OS情况。

### 5.2 前线治疗

TROP-2 ADC在NSCLC前线治疗研究的探索通常结合免疫治疗。ADC通过触发免疫原性细胞死亡、抗体依赖性细胞介导的细胞毒性和树突状细胞活化从而对免疫疗法起到补充作用^[41]^。EVOKE-02研究显示无论PD-L1表达水平如何，戈沙妥珠单抗联合帕博利珠单抗在初治患者中均能获得较为理想的早期缓解。TROPION-Lung02研究显示Dato-DXd联合帕博利珠单抗（±铂类化疗）在AGAs阴性NSCLC初治及经治患者中的ORR较高。其他如TROPION-Lung04和SKB264联合KL-A167的研究也证实了TROP-2 ADCs与免疫治疗联用的有效性。目前，多个3期研究正在展开，EVOKE-03、TROPION-Lung07、TROPION-Lung08研究和MK-2870-007研究拟探索戈沙妥珠单抗、Dato-DXd或SKB264联合帕博利珠单抗的疗效。TROPION-Lung10和AVANZAR研究拟探索Dato-DXd与Rilvegostomig或度伐利尤单抗的联合疗效。这些研究旨在探索ADC-免疫联合治疗方案对提高治疗效果和患者生存期的潜力。

### 5.3 不同人群的治疗

在伴有AGAs改变的NSCLC患者中，TROPION-Lung01研究非鳞癌患者的亚组显示，Dato-DXd单药治疗相较于多西他赛能够更有效地改善患者生存情况。TROPION-Lung05试验针对AGAs患者发现，EGFR突变患者的ORR达到43.6%，亚洲队列也表现出类似水平。此外，SKB264在TKIs耐药的EGFR突变NSCLC患者中也展现了良好疗效。目前，包括VELOCITY-Lung、ORCHARD、TROPION-Lung14和TROPION-Lung15等研究正在进行，探索不同TROP-2 ADC联合奥希替尼等免疫治疗的应用前景。同时，SKB264-III-09研究也在探索SKB264和培美曲塞联合治疗在AGAs患者中的效果。

对于伴有脑转移的NSCLC，前述基础研究已显示TROP-2 ADC在突破传统药物血脑屏障限制方面展现出独特的优势。TROPION-Lung05研究^[31]^数据表明，无论患者基线时是否伴有脑转移，Dato-DXd均能带来临床获益，尤其是在脑转移患者中的颅内缓解率达到22%，表明TROP-2 ADC有望成为未来治疗脑转移NSCLC的重要手段，有待进一步深入研究。

### 5.4 ADC药物的优化

TROP-2 ADCs的结构优化，尤其是连接体和有效载荷的改进，将在提升药物疗效和安全性方面发挥关键作用。Dato-DXd采用了独特的四肽连接体，SKB264的连接体相较于戈沙妥珠单抗也更具稳定性。此外，除了优化组分，尚有新型选择，包括使用双特异性抗体增强肿瘤特异性，使用小分子药物代替抗体以提高穿透力，以及尝试其他有效载荷，如非细胞毒性有效载荷相比传统的细胞毒性有效载荷具有潜在优势，包括降低对健康细胞的毒性和提高对癌细胞的特异性。期待未来新一代TROP-2 ADC进一步提升针对晚期NSCLC患者的治疗效果。

### 5.5 ADC在其他肺癌领域的开发

2023年欧洲肿瘤内科学会大会上公布了戈沙妥珠单抗治疗小细胞肺癌的初步研究结果，研究纳入了既往接受过不超过1线铂类化疗和抗PD-1/PD-L1治疗后进展的广泛期小细胞肺癌患者，疗效分析显示ORR达到了29%^[42]^。这为TROP-2 ADC治疗进一步扩大应用范围提供了启示。

## References

[1] Duma N, Santana-Davila R, Molina JR. Non-small cell lung cancer: epidemiology, screening, diagnosis, and treatment. Mayo Clin Proc, 2019, 94(8): 1623-1640. doi: 10.1016/j.mayocp.2019.01.013 31378236

[2] Zhou Y, Yang Y, Yang C, et al. Epidermal growth factor receptor (EGFR) mutations in non-small cell lung cancer (NSCLC) of Yunnan in southwestern China. Oncotarget, 2017, 8(9): 15023-15033. doi: 10.18632/oncotarget.14706 28107191 PMC5362464

[3] Jia B, Lv C, Chang JH, et al. Progressions in targeted therapy for non-small-cell lung carcinoma with uncommon mutations. Zhonghua Yixue Zazhi, 2022, 102(13): 969-976.

[4] Yang JC, Lee DH, Lee JS, et al. Phase III KEYNOTE-789 study of pemetrexed and platinum with or without pembrolizumab for tyrosine kinase inhibitor-resistant, EGFR-mutant, metastatic nonsquamous non-small cell lung cancer. J Clin Oncol, 2024, 42(34): 4029-4039. doi: 10.1200/JCO.23.02747 39173098 PMC11608596

[5] Li Z, Lu S. Who should receive the chemotherapy-free combination of nivolumab plus ipilimumab as the first-line treatment of advanced non-small-cell lung cancer?. J Clin Oncol, 2023, 41(6): 1172-1175. doi: 10.1200/JCO.22.02278 36623229

[6] Wu YL, Lu S, Cheng Y, et al. Nivolumab versus docetaxel in a predominantly Chinese patient population with previously treated advanced NSCLC: CheckMate 078 randomized phase III clinical trial. J Thorac Oncol, 2019, 14(5): 867-875. doi: 10.1016/j.jtho.2019.01.006 30659987

[7] Zhou C, Huang D, Fan Y, et al. Tislelizumab versus docetaxel in patients with previously treated advanced NSCLC (RATIONALE-303): A phase 3, open-label, randomized controlled trial. J Thorac Oncol, 2023, 18(1): 93-105. doi: 10.1016/j.jtho.2022.09.217 36184068

[8] Inamura K, Yokouchi Y, Kobayashi M, et al. Association of tumor TROP 2 expression with prognosis varies among lung cancer subtypes. Oncotarget, 2017, 8(17): 28725-28735. doi: 10.18632/oncotarget.15647 28404926 PMC5438686

[9] Bardia A, Messersmith WA, Kio EA, et al. Sacituzumab govitecan, a Trop-2-directed antibody-drug conjugate, for patients with epithelial cancer: final safety and efficacy results from the phase I/II IMMU-132-01 basket trial. Ann Oncol, 2021, 32(6): 746-756. doi: 10.1016/j.annonc.2021.03.005 33741442

[10] Okajima D, Yasuda S, Maejima T, et al. Datopotamab deruxtecan, a novel TROP2-directed antibody-drug conjugate, demonstrates potent antitumor activity by efficient drug delivery to tumor cells. Mol Cancer Ther, 2021, 20(12): 2329-2340. doi: 10.1158/1535-7163.MCT-21-0206 34413126 PMC9398094

[11] Cheng Y, Yuan X, Tian Q, et al. Preclinical profiles of SKB264, a novel anti-TROP2 antibody conjugated to topoisomerase inhibitor, demonstrated promising antitumor efficacy compared to IMMU-132. Front Oncol, 2022, 12: 951589. doi: 10.3389/fonc.2022.951589 36620535 PMC9817100

[12] Parisi C, Mahjoubi L, Gazzah A, et al. TROP-2 directed antibody-drug conjugates (ADCs): The revolution of smart drug delivery in advanced non-small cell lung cancer (NSCLC). Cancer Treat Rev, 2023, 118: 102572. doi: 10.1016/j.ctrv.2023.102572 37230055

[13] Goldenberg DM, Stein R, Sharkey RM. The emergence of trophoblast cell-surface antigen 2 (TROP-2) as a novel cancer target. Oncotarget, 2018, 9(48): 28989-29006. doi: 10.18632/oncotarget.25615 29989029 PMC6034748

[14] Stoyanova T, Goldstein AS, Cai H, et al. Regulated proteolysis of Trop 2 drives epithelial hyperplasia and stem cell self-renewal via β-catenin signaling. Genes Dev, 2012, 26(20): 2271-2285. doi: 10.1101/gad.196451.112 23070813 PMC3475800

[15] Trerotola M, Li J, Alberti S, et al. Trop-2 inhibits prostate cancer cell adhesion to fibronectin through the β1 integrin-RACK1 axis. J Cell Physiol, 2012, 227(11): 3670-3677. doi: 10.1002/jcp.24074 22378065 PMC3369113

[16] Levy BP, Felip E, Reck M, et al. TROPION-Lung08: phase III study of datopotamab deruxtecan plus pembrolizumab as first-line therapy for advanced NSCLC. Future Oncol, 2023, 19(21): 1461-1472. doi: 10.2217/fon-2023-0230 37249038

[17] Jones KJ, Suksomboon M rosenthal S, et al. Abstract 1911: Dato-DXd mediates anti-tumor activity in preclinical TROP2-expressing intracranial tumor model. Cancer Res, 2024, 84(6_Supplement): 1911. doi: 10.1158/1538-7445.AM2024-1911

[18] Abuhelwa Z, Alloghbi A, Nagasaka M. A comprehensive review on antibody-drug conjugates (ADCs) in the treatment landscape of non-small cell lung cancer (NSCLC). Cancer Treat Rev, 2022, 106: 102393. doi: 10.1016/j.ctrv.2022.102393 35472631 PMC12582167

[19] Goldenberg DM, Cardillo TM, Govindan SV, et al. Trop-2 is a novel target for solid cancer therapy with sacituzumab govitecan (IMMU-132), an antibody-drug conjugate (ADC). Oncotarget, 2015, 6(26): 22496-22512. doi: 10.18632/oncotarget.4318 26101915 PMC4673178

[20] Nagai Y, Oitate M, Shiozawa H, et al. Comprehensive preclinical pharmacokinetic evaluations of trastuzumab deruxtecan (DS-8201a), a HER2-targeting antibody-drug conjugate, in cynomolgus monkeys. Xenobiotica, 2019, 49(9): 1086-1096. doi: 10.1080/00498254.2018.1531158 30351177

[21] Paz-Ares LG, Juan-Vidal O, Mountzios GS, et al. Sacituzumab govitecan versus docetaxel for previously treated advanced or metastatic non-small cell lung cancer: the randomized, open-label phase III EVOKE-01 study. J Clin Oncol, 2024, 42(24): 2860-2872. doi: 10.1200/JCO.24.00733 38843511 PMC11328920

[22] Patel JD, Cho BC, Cobo M, et al. Sacituzumab govitecan (SG) + pembrolizumab (pembro) in first-line (1L) metastatic non-small cell lung cancer (mNSCLC) with PD-L1 ≥ 50%: Cohort A of EVOKE-02. J Clin Oncol, 2024, 42(16_suppl): 8592. doi: 10.1200/JCO.2024.42.16_suppl.8592

[23] Cho BC, editor. OA05.04 Sacituzumab govitecan + Pembrolizumab in 1L metastatic non-small cell lung cancer: preliminary results of the EVOKE-02 study. 2023 World Conference on Lung Cancer; 10 Sep 2023; Singapore.

[24] Ahn MJ, Cho BC, Goto Y, et al. 552P TROPION-Lung05: Datopotamab deruxtecan (Dato-DXd) in Asian patients (pts) with previously treated non-small cell lung cancer (NSCLC) with actionable genomic alterations (AGAs). Ann Oncol, 2023, 34: S1684-S1685. doi: 10.1016/j.annonc.2023.10.630

[25] Shimizu T, Kitazono S, Garon EB, et al. O13-6 Japanese subgroup analysis of datopotamab deruxtecan in NSCLC cohort of TROPION-PanTumor01, a phase 1 study. Ann Oncol, 2022, 33: S476. doi: 10.1016/j.annonc.2022.05.045

[26] Sun Y, Xiao Z, Cheng Y, et al. Datopotamab deruxtecan (Dato-DXd) in Chinese patients (pts) with advanced or metastatic non-small cell lung cancer (NSCLC): Results from the phase 1/2 TROPION-PanTumor02 study. J Clin Oncol, 2024, 42(16_suppl): 8548. doi: 10.1200/JCO.2024.42.16_suppl.8548

[27] Ahn MJ, Tanaka K, Paz-Ares L, et al. Datopotamab deruxtecan versus docetaxel for previously treated advanced or metastatic non-small cell lung cancer: the randomized, open-label phase III TROPION-Lung01 study. J Clin Oncol, 2024, JCO2401544. Epub ahead of print. doi: 10.1200/JCO-24-01544 PMC11771353 39250535

[28] Planchard D, Cozic N, Wislez M, et al. ICARUS-LUNG01: A phase 2 study of datopotomab deruxtecan (Dato-DXd) in patients with previously treated advanced non-small cell lung cancer (NSCLC), with sequential tissue biopsies and biomarkers analysis to predict treatment outcome. J Clin Oncol, 2024, 42(16_suppl): 8501. doi: 10.1200/JCO.2024.42.16_suppl.8501

[29] Papadopoulos KP. 1314MO TROPION-Lung05: Datopotamab deruxtecan (Dato-DXd) in previously treated non-small cell lung cancer (NSCLC) with actionable genomic alterations (AGAs). Ann Oncol, 2023, 34: S755-S756. doi: 10.1016/j.annonc.2023.09.2348

[30] Paz-Ares L. 1314MO TROPION-Lung05: Datopotamab deruxtecan (Dato-DXd) in previously treated non-small cell lung cancer (NSCLC) with actionable genomic alterations (AGAs). European Society for Medical Oncology; 2023 21 Oct 2023; Madrid, Spain.

[31] Lisberg A, Ahn MJ, Kitazono S, et al. Intracranial efficacy of datopotamab deruxtecan (Dato-DXd) in patients (pts) with previously treated advanced/metastatic non-small cell lung cancer (a/m NSCLC) with actionable genomic alterations (AGA): Results from TROPION-Lung05. J Clin Oncol, 2024, 42(16_suppl): 8593. doi: 10.1200/JCO.2024.42.16_suppl.8593

[32] Levy BP, Paz-Ares LG, Su WC, et al. Datopotamab deruxtecan (Dato-DXd) plus pembrolizumab (pembro) with or without platinum chemotherapy (Pt-CT) as first-line (1L) therapy for advanced non-small cell lung cancer (aNSCLC): Subgroup analysis from TROPION-Lung02. J Clin Oncol, 2024, 42(16_suppl): 8617. doi: 10.1200/JCO.2024.42.16_suppl.8617

[33] Goto Y, Su WC, Levy BP, et al. TROPION-Lung02: Datopotamab deruxtecan (Dato-DXd) plus pembrolizumab (pembro) with or without platinum chemotherapy (Pt-CT) in advanced non-small cell lung cancer (aNSCLC). J Clin Oncol, 2023, 41(16_suppl): 9004. doi: 10.1200/JCO.2023.41.16_suppl.9004

[34] Papadopoulos KP, Bruno D, Kitazono S, et al. Datopotamab deruxtecan (Dato-DXd) + Durvalumab ± Carboplatin in advanced/mNSCLC: Initial results from phase 1b TROPION-Lung04. J Thorac Oncol, 2023, 18(11): S55. doi: 10.1016/j.jtho.2023.09.043

[35] Fang W, Cheng Y, Chen Z, et al. Abstract CT247: Updated efficacy and safety of anti-TROP2 ADC SKB264 (MK-2870) for previously treated advanced NSCLC in phase 2 study. Cancer Res, 2024, 84(7_Supplement): CT247. doi: 10.1158/1538-7445.AM2024-CT247

[36] Fang W, Wang Q, Cheng Y, et al. Sacituzumab tirumotecan (SKB264/MK-2870) in combination with KL-A167 (anti-PD-L1) as first-line treatment for patients with advanced NSCLC from the phase II OptiTROP-Lung01 study. J Clin Oncol, 2024, 42(16_suppl): 8502. doi: 10.1200/JCO.2024.42.16_suppl.8502

[37] King GT, Eaton KD, Beagle BR, et al. A phase 1, dose-escalation study of PF-06664178, an anti-Trop-2/Aur 0101 antibody-drug conjugate in patients with advanced or metastatic solid tumors. Invest New Drugs, 2018, 36(5): 836-847. doi: 10.1007/s10637-018-0560-6 29333575 PMC7519583

[38] Marathe O, editor. 689P - DB-1305 (a Trop-2 targeted antibody-drug-conjugate [ADC]) in patients (pts) with advanced solid tumors: Preliminary clinical results from the phase (Ph) I/IIa study. European Society for Medical Oncology; 21 Oct 2023; Madrid, Spain.

[39] Fang W, Cheng Y, Chen Z, et al. SKB264 (TROP2-ADC) for the treatment of patients with advanced NSCLC: Efficacy and safety data from a phase 2 study. J Clin Oncol, 2023, 41(16_suppl): 9114. doi: 10.1200/JCO.2023.41.16_suppl.9114

[40] Auclin E, Benitez-Montanez J, Tagliamento M, et al. Second-line treatment outcomes after progression from first-line chemotherapy plus immunotherapy in patients with advanced non-small cell lung cancer. Lung Cancer, 2023, 178: 116-122. doi: 10.1016/j.lungcan.2023.02.002 36812760

[41] Passaro A, Jänne PA, Peters S. Antibody-drug conjugates in lung cancer: recent advances and implementing strategies. J Clin Oncol, 2023, 41(21): 3747-3761. doi: 10.1200/JCO.23.00013 37224424

[42] Dowlati A, Cervantes A, Babu S, et al. 1990MO Sacituzumab govitecan (SG) as second-line (2L) treatment for extensive stage small cell lung cancer (ES-SCLC): Preliminary results from the phase II TROPiCS-03 basket trial. Ann Oncol, 2023, 34: S1061-S1062.

[43] Borghaei H, Waqar SN, Bruno DS, et al. TROPION-Lung04: phase 1b, multicenter study of datopotamab deruxtecan (Dato-DXd) in combination with immunotherapy ± carboplatin in advanced/metastatic non-small cell lung cancer (mNSCLC). J Clin Oncol, 2023, 41(16_suppl): TPS3158. doi: 10.1200/JCO.2023.41.16_suppl.TPS3158

[44] Chen YF, Xu YY, Shao ZM, et al. Resistance to antibody-drug conjugates in breast cancer: mechanisms and solutions. Cancer Commun (Lond), 2023, 43(3): 297-337. doi: 10.1002/cac2.12387 36357174 PMC10009672

[45] The Society of Chemotherapy, China Anti-Cancer Association, Committee of Neoplastic Supportive-Care (CONS), China Anti-Cancer Association. Consensus on the clinical diagnosis, treatment, and prevention of chemotherapy-induced thrombocytopenia in China (2019 version). Zhongguo Zhongliu Linchuang, 2019, 46(18): 923-929.

[46] Professional Committee on Clinical Research of Oncology Drugs, Chinese Anti-Cancer Association, Expert Committee for Monitoring the Clinical Application of Antitumor Drugs, Breast Cancer Expert Committee of National Cancer Quality Control Center, et al. Expert consensus on the clinical application of antibody drug conjugates in the treatment of malignant tumors (2023 edition). Zhonghua Zhongliu Zazhi, 2023, 45(9): 741-762. 37460440 10.3760/cma.j.cn112152-20220713-00489

[47] Anticancer Drug-induced Interstitial Lung Disease Management Group. Expert consensus on the diagnosis and treatment of anticancer drug-induced interstitial lung disease. Zhonghua Zhongliu Zazhi, 2022, 44(7): 693-702. 35880334 10.3760/cma.j.cn112152-20220412-00244

[48] ASMC in CSCO, SCRC. Expert consencus on diagnosis,prevention and treatment of acute oral mucositis induced by anti-tumor therapy. Linchuang Zhongliuxue Zazhi, 2021, 26(5): 449-459.

[49] Breast Cancer Group, Branch of Oncologist, Chinese Medical Doctor Association; International Medical Exchange Society, Chinese Anti-Cancer Association. Chinese expert consensus of antibody-drug conjugate toxicity management for breast cancer. Zhonghua Zhongliu Zazhi, 2022, 44(9): 913-927. 36164692 10.3760/cma.j.cn112152-20220521-00360

